# The Bees among Us: Modelling Occupancy of Solitary Bees

**DOI:** 10.1371/journal.pone.0164764

**Published:** 2016-12-02

**Authors:** J. Scott MacIvor, Laurence Packer

**Affiliations:** 1 Department of Biological Sciences, University of Toronto Scarborough, Toronto, Canada; 2 Biology Department, York University, Toronto, Canada; University of Guelph, CANADA

## Abstract

Occupancy modelling has received increasing attention as a tool for differentiating between true absence and non-detection in biodiversity data. This is thought to be particularly useful when a species of interest is spread out over a large area and sampling is constrained. We used occupancy modelling to estimate the probability of three phylogenetically independent pairs of native—introduced species [*Megachile campanulae* (Robertson)*—Megachile rotundata* (Fab.), *Megachile pugnata* Say—*Megachile centuncularis* (L.), *Osmia pumila* Cresson—*Osmia caerulescens* (L.)] (Apoidea: Megachilidae) being present when repeated sampling did not always find them. Our study occurred along a gradient of urbanization and used nest boxes (bee hotels) set up over three consecutive years. Occupancy modelling discovered different patterns to those obtained by species detection and abundance-based data alone. For example, it predicted that the species that was ranked 4^th^ in terms of detection actually had the greatest occupancy among all six species. The native *M*. *pugnata* had decreased occupancy with increasing building footprint and a similar but not significant pattern was found for the native *O*. *pumila*. Two introduced bees (*M*. *rotundata* and *M*. *centuncularis*), and one native (*M*. *campanulae*) had modelled occupancy values that increased with increasing urbanization. Occupancy probability differed among urban green space types for three of six bee species, with values for two native species (*M*. *campanulae* and *O*. *pumila*) being highest in home gardens and that for the exotic *O*. *caerulescens* being highest in community gardens. The combination of occupancy modelling with analysis of habitat variables as an augmentation to detection and abundance-based sampling is suggested to be the best way to ensure that urban habitat management results in the desired outcomes.

## Introduction

### Detection probability

A persistent problem with understanding the results of repeated biodiversity surveys is that of false absence: when a species is present at the site but not detected in a sample [1]. This limitation is more readily assessed with repeated sampling at multiple locations or times where non-detections are interspersed among instances of detection; these patterns permit the estimation of a detection probability per species and the proportion of non-detection that actually indicates true absences [1]. Interpreting non-detection as absence will underestimate a species’ temporal and/or spatial distribution [2,3]. This can decrease the accuracy of habitat models [4] and may weaken the effectiveness of wildlife management recommendations [3,5–9]. The probability of detecting a species (*p*) is related to species occupancy (Ψ), a state variable that estimates the proportion of sites that are occupied by a species, whether or not it was detected in surveying [1]. Occupancy, as estimated with Ψ, does not consider abundance, only the presence or absence of a species at a site during sampling [10–12].

Occupancy models incorporate both Ψ and *p* and are especially useful for interpreting survey data of species that are difficult to sample, and/or where populations are common and/or widespread but extensive sampling is prohibitive in either cost or time [3]. These models permit the assessment of likelihood of the species being present at sites where the species of interest was not detected [13,14]. In sum, occupancy modelling should provide a more reliable picture of a species’ presence among a series of samples irrespective of the proportion of times it was actually detected in sampling [2,14–18].

Welsh *et al*. (2013) argued that results from occupancy models can be highly variable depending on the number of individuals surveyed and that interpreting them can be as misleading as ignoring non-detection in abundance-based studies [9]. Here we present the results of occupancy modelling on the six most common bee species from trap nest surveys in a large urban landscape. Three are native and three introduced. We demonstrate that conclusions based on occupancy models are often different from those based upon sampled detection. Thus, we find that occupancy models provide additional insights into the determinants of bee occurrences in the urban milieu. Further, we argue that failure to detect a species in a sample when it might have been present should be acknowledged in ecological studies, and our data supports the notion that occupancy modelling produces meaningful results by partitioning true absence from false non-detection. Occupancy modelling should be added to the toolbox used by urban ecologists as it has implications for biodiversity management and planning of complementary urban green spaces, such as private home gardens or green roofs that can be difficult to access and sample repeatedly [19].

### Bees

Bees are essential pollinators in most terrestrial landscapes for both agricultural crops [20–22] and wild plants [23]. Consequently, they have been studied using a variety of abundance-based sampling techniques [24–28]. Discovery of bee declines has resulted in increased monitoring, conservation action, and public awareness [29–36].

Irrespective of sample size, bee surveys often contain many species represented as singletons [37,38] and it is difficult to measure species diversity accurately when many are rare [25]. Also, as bees forage away from their nest [39–41], their presence in samples may not be indicative of habitat suitability at the sample site per se. For example, individual bees may be just ‘passing through’ the habitat under investigation as they fly between their nest and floral resources [26].

Suitable foraging habitat for bees in urban landscapes is fragmented and heterogeneous, consisting of a mix of small and large patches supporting a diverse array of flowering plant species and varieties [42–44]. These patches can support urban pollinator diversity [45,46] but perhaps unsurprisingly, bee diversity generally declines with increasing urbanization [47,48] or exhibits no significant change [49,50]. However, one group, the cavity-nesting bees, seems to have a disproportionally higher representation in urban areas (except those where impervious surfaces extend beyond 50% of land cover) [43,51]. This is presumably because suitable nest sites are more numerous due to increased numbers of cut plant stems, woody debris, home gardening structures (i.e. holes in garden sheds or fences) [43], and nest boxes [52]. Nest boxes are inexpensive to build and easy to monitor [19]. As they sample nests directly, nest boxes can be used to assess habitat quality because they do not include taxa that are merely passing through the area [53]. They are particularly useful for sampling large numbers of sites simultaneously as they are put out before seasonal bee activity begins and taken down after it ends.

In this study we use occupancy modelling to investigate differences in populations of native and introduced cavity-nesting bees in nest boxes at sites >250m apart throughout a large city over three years. We compare results among introduced and native species because: i) introduced bees can have negative impacts on both native bees [54–57] and pollination networks [58]; ii) they are increasingly represented in surveys of wild bees [26,51,59–61]; and iii) because introduced bees have been moved from one continent to another by human activity, a greater level of synanthropic adaptation might be found among them [62,63]. Consequently, our first hypothesis is that introduced species would have greater occupancy probabilities than native species. Detected bee species diversity declines towards those areas of cities where the proportion of buildings and impervious surfaces are highest [43,44,51]. Thus, our second hypothesis was that occupancy probabilities for all bees examined would decline with increasing urbanization as determined by the proportion of building footprint surrounding a site.

## Methods

### Sampling

Nest boxes were set up at sites throughout the city of Toronto and the surrounding region each year from 2011 to 2013 inclusive (S1 Fig). Four urban green space types (“type”) were differentiated: home gardens, community gardens, urban parks, and building rooftops. Permission was granted to sample in urban parks from the Toronto and Region Conservation Authority and city of Toronto park staff. Permission was also granted from homeowners, community gardeners, and building managers to sample in home gardens, community gardens and on rooftops, respectively. Home gardens were either front- or backyards occurring on privately owned property and maintained by a homeowner. Community gardens occupied a central location: i.e. a neighbourhood park, the grounds of an apartment complex, or a power line (hydro) corridor, where groups of people garden collectively. Urban parks were sites contained within the boundaries of named parks as designated by the City of Toronto and the Toronto and Region Conservation Authority (TRCA). These are usually grassy areas with sparse tree cover but usually with planted flowerbeds around the edges or along paths [64]. Building rooftop sites were atop single buildings upon which vegetation (i.e. planters, green roofs) had been installed [65]. Green roofs are increasingly common in Toronto where they are mandatory on new buildings of certain types [66].

Each nest box was constructed from a 30 cm piece of recyclable PVC pipe of 10 cm diameter with one end fitted with a covered pipe cap, the other with an open faceplate of insulation board with 30 cardboard tubes inserted. Cardboard tubes were of three different internal diameters (10 of each of: 3.4mm, 5.5mm and 7.6mm) to accommodate bees of different sizes and were each plugged with papier-mâché at the capped end of the pipe [19]. Nest boxes were set up facing southeast and attached using zip-ties to fixed features in the landscape. These included fence posts, exposed tree limbs, or other forms of urban infrastructure so that each nest box would not move, and was above the maximum height of any immediately surrounding vegetation (>1.2m off the ground).

Each year, all nest boxes were set up in April and taken down in October. Once recovered, the cardboard tubes were opened and the contents of each recorded. Altogether samples were taken from 199 sites. Bees were kept in cold storage (October-March) before transfer to a growth chamber where they were incubated and reared to adulthood for identification. From a total of 36 bee species found, six megachilids were selected for occupancy modelling because they were common and widespread [3] (Table 1). For each of the six species, the total number of brood cells constructed was recorded as the abundance per site, and the total number of nesting tubes colonized was also recorded from each sampling site/year. Since the differences in response to urbanization between native and introduced bees might be phylogenetically constrained, we grouped the native and introduced bees [67,68] into pairs that exhibit reciprocal monophyly. Based upon available phylogenies [69], the species pairs are as follows (native species first within each pair): *Megachile campanulae* (Robertson) + *M*. *rotundata* (Fab.); *M*. *pugnata* Say + *M*. *centuncularis* (L.); *Osmia pumila* Cresson + *O*. *caerulescens* L.

**Table 1:** A list of the six bee species studied and the model equation used to fit the presence-absence data for each, as collected over the three-year study period. The nesting tube diameters used (the preferred diameter in bold) and the observed frequency from the sample across all sites are also included.

Species	Nest Diameter	Actual Site Occupancy	Model Equation
Native			
*Megachile campanulae* (Robertson)	**5.5**, 7.6	0.286	Ψ(site),*p*(.)
*Megachile pugnata* Say	5.5, **7.6**	0.045	Ψ(foot,site),*p*(site)
*Osmia pumila* Cresson	**3.4**, 5.5	0.322	Ψ(site),*p*(.)
Introduced			
*Megachile rotundata* (Fabricius)	3.4, **5.5**, 7.6	0.337	Ψ(site),*p*(foot,site)
*Megachile centuncularis* (Linnaeus)	5.5, **7.6**	0.176	Ψ(foot),*p*(.)
*Osmia caerulescens* Linnaeus	3.4, **5.5**	0.342	Ψ(site),*p*(foot,site)

### Analysis

City of Toronto spatial reference data shapefiles (RMSI, Toronto, Ontario) were examined using geospatial tools in ArcGIS v.10 (ESRI, Toronto, Canada). To determine site variables potentially impacting bee presence, the proportion of building footprint (m^2^) (hereafter referred to as ‘foot’) within a 300m radius around each site was determined. We used this radius for two reasons: i) local habitat structure has a greater impact upon bees than does landscape-scale structure [39] and ii) small to medium-sized solitary bees that use nest boxes rarely travel further than 300m from their nest [40,41]. The proportion of area covered by buildings is a good indicator of urbanization [70] and is applicable citywide across different land use types [71], consequently, building footprint was summed for all building types. This metric was extracted using the buffer and clip tools in ArcGIS within the 300m radius surrounding each site. Z-scores were calculated to standardize the building footprint values prior to statistical testing.

Presence/absence data for the six bee species were recorded from nest boxes at each site for each year and analysed using the program, PRESENCE [3]. This program permits the user to estimate the proportion of sites occupied (Ψ) and the detection probability per site (*p*) for specific taxa in relation to different site variables. To interpret Ψ from each site over the three years, each year was considered a single sample, and a single season model in PRESENCE was used to examine each species independently. Nest boxes provided data on annual detection of bee species; the bees are collected once per year after the nest box is opened, and not returned to the site from which they were collected, hence, each site/year is a closed sample in which a species is recorded as either present or absent. As a result, data for each species were collapsed into the single-season feature in PRESENCE, which is conventionally used to fit multiple samples from a single season, rather than a multiple-season model requiring multiple samples for each season [3]. To further comply with the model assumptions, we had three consecutive samples per site, which is the minimum required to eliminate biases associated with false absences [4,5] and allow for interpretation of spatial occupancy patterns not apparent from detection or abundance data alone [1]. All possible combinations of urban green space site type (‘site’) and building footprint (‘foot’) were fit to Ψ and *p* parameters and each model equation was applied to the presence data for each species separately (S1 Table). The model of best fit was determined using AIC model selection [72] for each species (Table 1).

To quantify any uncertainty in our occupancy estimates and assess model convergence, Markov chain Monte Carlo (MCMC) algorithms were implemented using the Gibbs sampling program JAGS and the accompanying program rjags [73] in RStudio v0.98 [74]. For each model, we ran three chains with 10,000 iterations each for 30,000 total. We used the Gelman-Rubin convergence diagnostic to assess model convergence with potential scale reduction factor (PSRF) values approaching 1 (and no higher than 1.1) considered acceptable [75].

**Fig 1:**
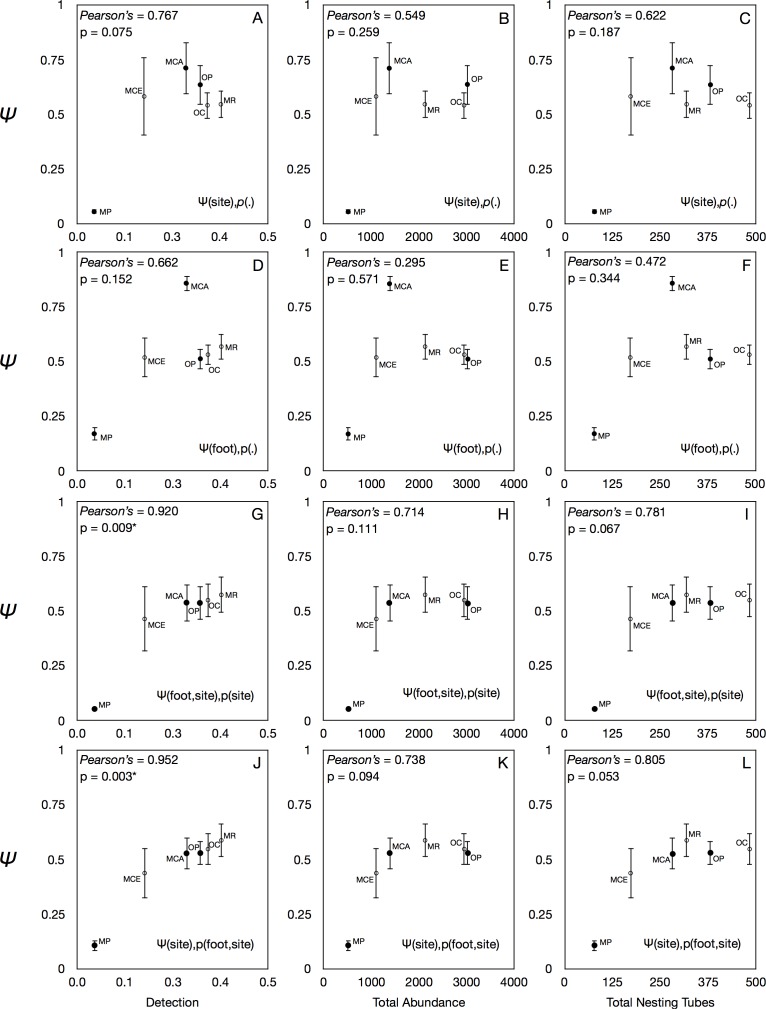
Rank correlations of species detection, abundance, and number of nesting tubes colonized against occupancy estimates for all species derived from the top four model equations. Top model equations were determined by AIC applied to each species individually (see Table 1). Plots A-C show results for Ψ estimates from model equation Ψ(site),*p*(.), D-F is Ψ(foot),*p*(.), G-I is Ψ(site),*p*(foot,site), J-L is Ψ(foot,site),*p*(site). An asterisk indicates significance at the α = 0.05 level. Native species are denoted with opaque circles and introduced species with open circles.

A Pearson’s correlation test was used to determine whether Ψ estimates were correlated with species detection, species abundance or the number of nesting tubes colonized over all sampling sites/years for all four models and for each of the six species (see Fig 1). Using estimates from the model equation Ψ(site),*p*(.), linear regression analysis (α = 0.05) was used to compare individual species’ Ψ, site abundance and the number of nesting tubes colonized against building footprint and the coefficients qualitatively compared among the six species. For each species, an analysis of variance (ANOVA) was used to test for significant differences in species’ Ψ among the four urban green space types defined.

## Results

**Fig 2:**
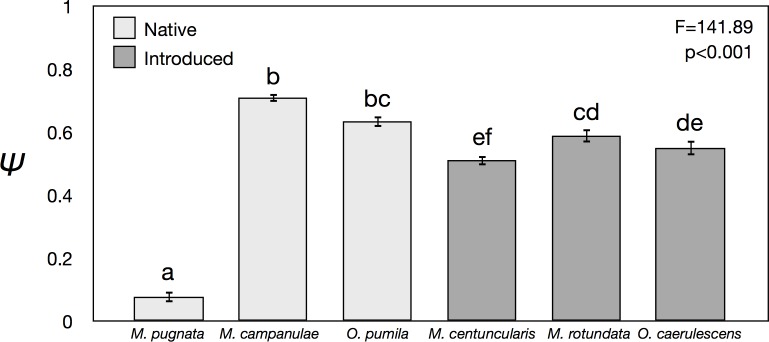
Occupancy probability scores for each of the six species using the model equation Ψ(site),*p*(.). Significant differences (α = 0.05) are indicated alphabetically. Native species in pale grey and introduced species in darker grey.

Among the six bee species examined, the ordering of species by predicted occupancy (Ψ) differed from that based upon actual detection, total abundance, and the number of nesting tubes colonized (Fig 1). Introduced *O*. *caerulescens* and *M*. *rotundata* and the native *O*. *pumila* were all detected at more sites and were more abundant than *M*. *campanulae* (Fig 1A), even though the Ψ for *M*. *campanulae* was significantly greater than that of all other species except *O*. *pumila* (Fig 2). Variances in the model estimates of Ψ were lower for each of the introduced species than for the native ones (Fig 1).

The model equations of best fit for interpreting data as determined by AIC selection are shown in Table 1. The Gelman-Rubin convergence diagnostic tests indicated that all top models for all species converged appropriately (S1 File) as each PSRF value calculated was equal to 1. Species detection was positively correlated with occupancy in two of the four top models, Ψ(site),*p*(foot,site) (Fig 1G) and Ψ(foot,site),*p*(site) (Fig 1J). There was also a moderately positive relationship between occupancy and the number of nesting tubes completed (Fig 1L). Species abundance was not correlated with Ψ estimates using the models of best fit for any species or for any of the other top model equations (Fig 1).

The model equations of best fit indicated that only Ψ of the native *M*. *pugnata* was negatively correlated with the proportion of building footprint surrounding the nesting site within a 300m radius (S2 Table). Increasing building footprint also led to a significant decline in the detected abundance and number of nesting tubes colonized by *O*. *pumila* (F = 1.986, p = 0.001 and F = 1.841, p = 0.003, respectively, S2 Table). No other significant differences were recorded for any other species, however two introduced bees (*M*. *rotundata* and *M*. *centuncularis*) and one native (*M*. *campanulae*) had occupancy values that increased with building footprint (S2 Table).

**Fig 3:**
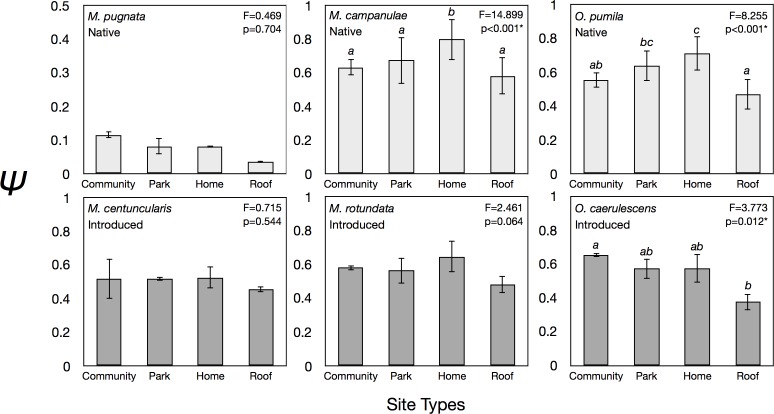
Mean occupancy probabilities of native and introduced bee species when grouped by site using the model equation Ψ(site),*p*(.). Community gardens (N = 14), building rooftops (N = 20), city parks (N = 43), and home gardens (N = 72).

The type of urban green space had a significant impact on Ψ of two natives (*M*. *campanulae* and *O*. *pumila*) and one introduced bee (*O*. *caerulescens*) (Fig 3). Both natives had Ψ greatest in home gardens while *O*. *caerulescens* had greater Ψ in community gardens compared to roofs, but not when compared to parks or home gardens (Fig 3). Differences in Ψ among site types for introduced *M*. *rotundata* approached significance, with home gardens exhibiting the highest estimates.

## Discussion

This study is the first to employ occupancy modelling as a tool to estimate patterns in bee detection and false absences. We found that estimates of occupancy probabilities provided additional details for each species that were not evident from interpretation of detection or abundance data alone (Fig 1). For example, the native *M*. *campanulae* had the greatest Ψ recorded among all six bees, i.e. it was predicted to be present at the most sites whether it was found in the nest boxes or not. However, *M*. *campanulae* ranked 4^th^ in detection, abundance, and the number of nesting tubes colonized. This illustrates the value of the additional data provided through occupancy modelling: even though *M*. *campanulae* was less abundant overall, it was predicted to be the most ubiquitous when non-detection was incorporated using occupancy modelling.

Occupancy probability estimates were more variable among native bees and more consistently high among introduced species (Fig 2). However, there was no indication that introduced bees exhibited higher Ψ than native bees overall, leading to rejection of our first hypothesis: that introduced bees would have higher occupancy than native bees. The two native bees (*M*. *campanulae* and *O*. *pumila*) exhibited the greatest occupancy probabilities among all those tested (Fig 2) despite two introduced species (*M*. *rotundata* and *O*. *caerulescens*) having higher site detection and total abundance (Fig 1). This example provides evidence that native bees may occur more broadly in urban environments [45,49,76] than is evident from abundance-based studies that find the most common species to be introduced ones [51,77,78].

### Gradient of urbanization

Overall, increasing urbanization, as determined by surrounding building footprint, was not correlated with Ψ for five of the six bee species. Increased building density had a strong negative impact on Ψ of one native (*M*. *pugnata*) which declined to 0 occupancy at sites with >30% building footprint (S1 File). Moreover, although the native *O*. *pumila* was the most abundant species and had high Ψ indicating it is widespread, its occupancy estimates declined with increasing building footprint. Nonetheless, based upon our data *in toto*, we reject the second hypothesis that Ψ by native species would decline more with building footprint than that of introduced bees.

Other studies of urban bee communities have found high bee diversity in areas of low to medium urbanization [50,79] and fewer species in the dense urban core [80] where the proportion of impervious surface is highest [81]. However, occupancy of three bee species, the native *M*. *campanulae* and the introduced *M*. *rotundata* and *M*. *centuncularis*, increased with building footprint.

### Urban green space type

Occupancy probabilities for native *M*. *campanulae* and *O*. *pumila* were significantly greater in home gardens compared to other urban green space types surveyed (except for no difference between home gardens and parks for *O*. *pumila*) (Fig 3). *Megachile campanulae* uses resins for nesting materials, obtaining them from a variety of trees including white pine [82], which are widely planted in home landscapes and other nearby urban green spaces. *Osmia pumila* also has its nesting material requirements (mud and masticated leaves) [83] widely distributed among our urban study sites. Our data support the view that home gardens are suitable for many native bees and may be critical in maintaining wild bee populations in urban landscapes [84,85].

Among introduced bees, *O*. *caerulescens* had Ψ that was higher in community gardens than elsewhere (Fig 3). Not surprisingly, community gardens have also been identified as hotspots for urban bee activity [86,87], as well as pollination services [88]. Occupancy probabilities for the other two introduced bees, *M*. *rotundata* and *M*. *centuncularis*, did not differ among site types, indicative of their flexibility to persist in a wide variety of urban green spaces, including vegetated rooftops. Although introduced bees can be effective pollinators of cultivated crops [89–91], they disproportionately visit introduced flowers and this could facilitate the outcompetition of native plants [92,93]. Nest boxes can contribute to the monitoring of introduced species that left unchecked could outcompete native bees with negative consequences for plant communities and pollination networks [19,59].

### Conclusion

This study illustrates the importance of including Ψ as a variable in biodiversity survey work: it yielded patterns that were biologically meaningful and different from those based upon detection and abundance data alone (Fig 1). Inclusion of environmental variables in more complex occupancy models could improve the precision of resulting estimates and provide a deeper explanation of patterns that increase the accuracy of monitoring or management of introduced species [94]. For example, the combination of nest box sampling with occupancy modelling will allow us to predict where introduced species exist but were not found during sampling. This may be particularly useful for determining areas of occupancy of aggressively spreading introduced species, such as *Megachile sculpturalis* [95,96].

Our data indicate that different bee species, even within the same nesting guild, thrive best in different urban green space types. This suggests that complementary and collaborative planning of such space could be specifically designed to foster native species. More research is required on the impacts of different management plans and conservation actions to ensure that ‘scaled up’ urban habitat alteration has positive outcomes [97,98]. We have found that occupancy modelling provides additional details that are not discovered with detection and abundance-based sampling and conclude that this approach should be incorporated into urban habitat management planning.

## Supporting Information

S1 Fig A map of the study area identifying the location of each site sampled. The type of urban green space is identified in the figure legend.
(PDF)

S1 File Occupancy estimates and standard error for the top four models for each species and site. As well as the proportion of impervious surface surrounding each site and the urban green space type (community gardens = 1, green roofs = 2, urban parks = 3, home gardens = 4).
(XLSX)

S1 Table Summary of model selection processes for each of the six bee species using Akiake’s Information Criterion (AIC). Ψ denotes the probability of a bee occupying a site when not detected, and *p* denotes the probability of being detected using a nest box when present at the site. The terms in parentheses indicate what factors are affecting each probability with a ‘.’ indicating the probability is constant across all states. ΔAIC is the relative difference in AIC values, *w* is the AIC model weight, -2*l* is twice the negative log-likelihood and *K* is the number of parameters in the model. For all models the same structure was maintained for the detection-related component of the model.
(DOCX)

S2 Table Statistical output from comparisons of occupancy probability per bee and the proportion of building footprint within a 300m radius around each site. Significant differences calculated using the Z-score of building footprint. Asterisk indicates significant difference within species.
(DOCX)
