# Supplementary figures and images for: The Bees among Us: Modelling Occupancy of Solitary Bees
Source: PLoS One. 2016 Dec 2;11(12):e0164764. doi: 10.1371/journal.pone.0164764 (PMC5135037; doi:10.1371/journal.pone.0164764)

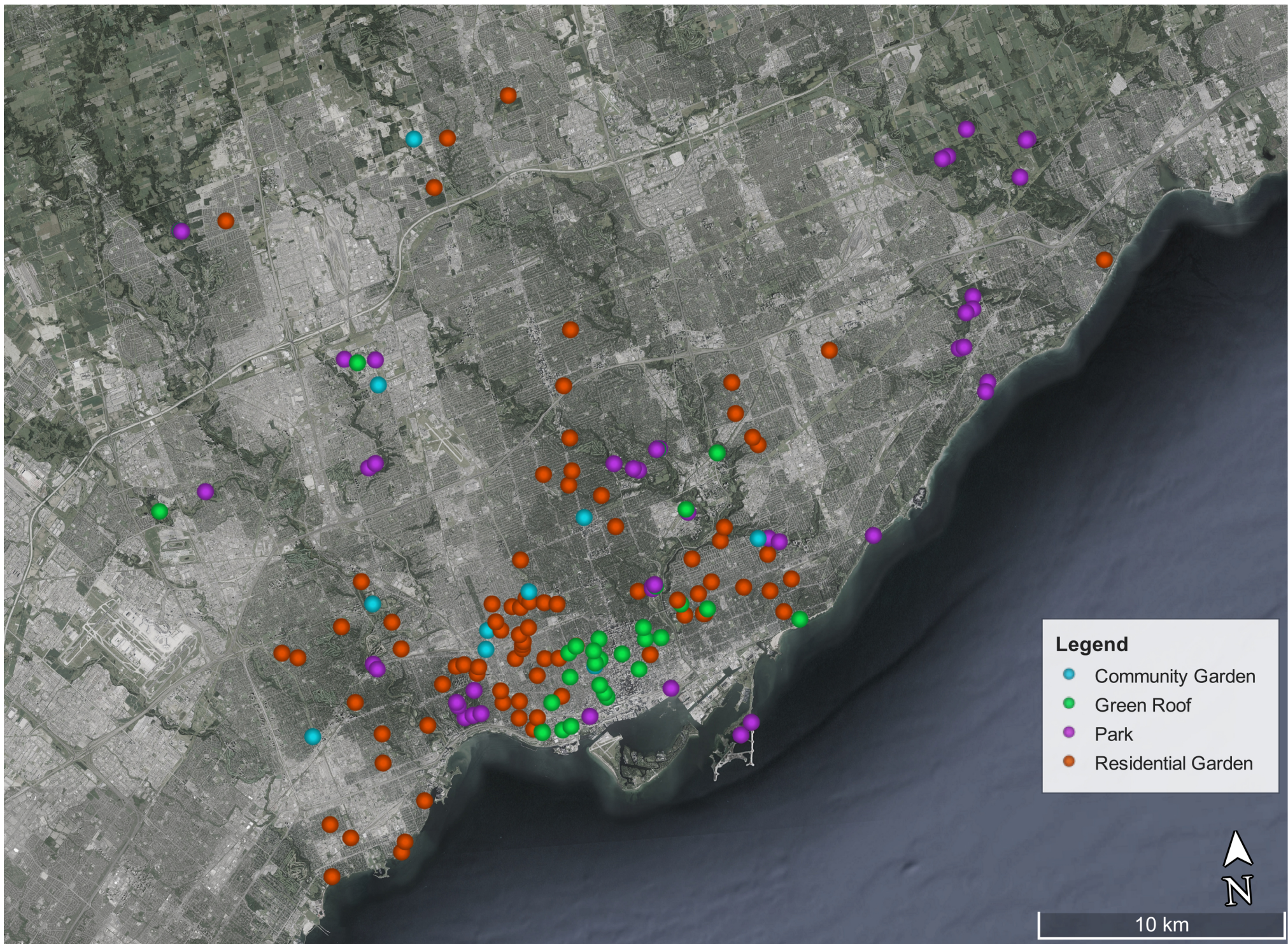

Supplement: S1 Fig — The type of urban green space is identified in the figure legend. (PDF) [file pone.0164764.s001.pdf]
