# Supplementary material for: The Bees among Us: Modelling Occupancy of Solitary Bees
Source: PLoS One. 2016 Dec 2;11(12):e0164764. doi: 10.1371/journal.pone.0164764 (PMC5135037; doi:10.1371/journal.pone.0164764)
Supplement: S1 Table — Ψ denotes the probability of a bee occupying a site when not detected, and p denotes the probability of being detected using a nest box when present at the site. The terms in parentheses indicate what factors are affecting each probability with a ‘.’ indicating the probability is constant across all states. ΔAIC is the relative difference in AIC values, w is the AIC model weight, -2l is twice the negative log-likelihood and K is the number of parameters in the model. For all models the same structure was maintained for the detection-related component of the model. (DOCX) [file pone.0164764.s003.docx]

S1 Table. Summary of model selection processes for each of the six bee species using Akiake’s Information Criterion (AIC). Ψ denotes the probability of a bee occupying a site when not detected, and *p* denotes the probability of being detected using a nest box when present at the site. The terms in parentheses indicate what factors are affecting each probability with a ‘.’ indicating the probability is constant across all states. ΔAIC is the relative difference in AIC values, *w* is the AIC model weight, -2*l* is twice the negative log-likelihood and *K* is the number of parameters in the model. For all models the same structure was maintained for the detection-related component of the model.

| Species | Model | AIC | ΔAIC | AIC weights | Model Likelihood | K | -2*LogLike |
| --- | --- | --- | --- | --- | --- | --- | --- |
| *M. campanulae* | Ψ(site),*p*(.) | 411.33 | 0.00 | 0.4605 | 1.0000 | 2 | 407.33 |
|  | Ψ(foot,site),*p*(.) | 412.05 | 0.72 | 0.3213 | 0.6977 | 3 | 406.05 |
|  | Ψ(.),*p*(.) | 413.48 | 2.15 | 0.1572 | 0.3413 | 2 | 409.48 |
|  | Ψ(foot),*p*(.) | 415.39 | 4.06 | 0.0605 | 0.1313 | 2 | 411.39 |
|  | Ψ(site),*p*(site) | 428.00 | 16.67 | 0.0001 | 0.0002 | 2 | 424.00 |
|  | Ψ(foot),*p*(site) | 428.02 | 16.69 | 0.0001 | 0.0002 | 2 | 424.02 |
|  | Ψ(.),*p*(site) | 428.08 | 16.75 | 0.0001 | 0.0002 | 2 | 424.08 |
|  | Ψ(foot),*p*(foot,site) | 428.77 | 17.44 | 0.0001 | 0.0002 | 3 | 422.77 |
|  | Ψ(foot,site),*p*(site) | 429.37 | 18.04 | 0.0001 | 0.0001 | 3 | 423.37 |
|  | Ψ(foot,site),*p*(foot,site) | 429.81 | 18.48 | 0.0000 | 0.0001 | 4 | 421.81 |
|  | Ψ(site),*p*(foot,site) | 429.87 | 18.54 | 0.0000 | 0.0001 | 3 | 423.87 |
|  | Ψ(.),*p*(foot,site) | 430.00 | 18.67 | 0.0000 | 0.0001 | 3 | 424.00 |
|  | Ψ(.),*p*(foot) | 450.17 | 38.84 | 0.0000 | 0.0000 | 2 | 446.17 |
|  | Ψ(site),*p*(foot) | 455.64 | 44.31 | 0.0000 | 0.0000 | 2 | 451.64 |
|  | Ψ(foot,site),*p*(foot) | 457.27 | 45.94 | 0.0000 | 0.0000 | 3 | 451.27 |
|  | Ψ(foot),*p*(foot) | 458.48 | 47.15 | 0.0000 | 0.0000 | 2 | 454.48 |
|  |  |  |  |  |  |  |  |
| Species | Model | AIC | ΔAIC | AIC weights | Model Likelihood | K | -2*LogLike |
| *O. pumila* | Ψ(site),*p*(.) | 456.96 | 0.00 | 0.5082 | 1.0000 | 2 | 452.96 |
|  | Ψ(foot,site),*p*(.) | 458.84 | 1.88 | 0.1985 | 0.3906 | 3 | 452.84 |
|  | Ψ(.),*p*(.) | 459.35 | 2.39 | 0.1538 | 0.3027 | 2 | 455.35 |
|  | Ψ(foot),*p*(.) | 459.63 | 2.67 | 0.1337 | 0.2632 | 2 | 455.63 |
|  | Ψ(.),*p*(site) | 468.89 | 11.93 | 0.0013 | 0.0026 | 2 | 464.89 |
|  | Ψ(site),*p*(site) | 468.98 | 12.02 | 0.0012 | 0.0025 | 2 | 464.98 |
|  | Ψ(foot),*p*(site) | 469.23 | 12.27 | 0.0011 | 0.0022 | 2 | 465.23 |
|  | Ψ(.),*p*(foot,site) | 470.84 | 13.88 | 0.0005 | 0.0010 | 3 | 464.84 |
|  | Ψ(site),*p*(foot,site) | 470.86 | 13.90 | 0.0005 | 0.0010 | 3 | 464.86 |
|  | Ψ(foot,site),*p*(site) | 470.91 | 13.95 | 0.0005 | 0.0009 | 3 | 464.91 |
|  | Ψ(foot),*p*(foot,site) | 471.23 | 14.27 | 0.0004 | 0.0008 | 3 | 465.23 |
|  | Ψ(foot,site),*p*(foot,site) | 472.85 | 15.89 | 0.0002 | 0.0004 | 4 | 464.85 |
|  | Ψ(.),*p*(foot) | 481.31 | 24.35 | 0.0000 | 0.0000 | 2 | 477.31 |
|  | Ψ(foot,site),*p*(foot) | 483.02 | 26.06 | 0.0000 | 0.0000 | 3 | 477.02 |
|  | Ψ(site),*p*(foot) | 485.37 | 28.41 | 0.0000 | 0.0000 | 2 | 481.37 |
|  | Ψ(foot),*p*(foot) | 488.53 | 31.57 | 0.0000 | 0.0000 | 2 | 484.53 |
|  |  |  |  |  |  |  |  |
| Species | Model | AIC | ΔAIC | AIC weights | Model Likelihood | K | -2*LogLike |
| *M. pugnata* | Ψ(foot,site),*p*(site) | 89.51 | 0.00 | 0.9098 | 1.0000 | 3 | 83.51 |
|  | Ψ(foot,site),*p*(foot) | 96.90 | 7.39 | 0.0226 | 0.0248 | 3 | 90.90 |
|  | Ψ(foot),*p*(foot,site) | 96.91 | 7.40 | 0.0225 | 0.0247 | 3 | 90.91 |
|  | Ψ(foot),*p*(site) | 97.00 | 7.49 | 0.0215 | 0.0236 | 2 | 93.00 |
|  | Ψ(foot),*p*(.) | 97.97 | 8.46 | 0.0132 | 0.0146 | 2 | 93.97 |
|  | Ψ(foot,site),*p*(foot,site) | 98.72 | 9.21 | 0.0091 | 0.0100 | 4 | 90.72 |
|  | Ψ(.),*p*(foot) | 105.12 | 15.61 | 0.0004 | 0.0004 | 2 | 101.12 |
|  | Ψ(.),*p*(.) | 105.57 | 16.06 | 0.0003 | 0.0003 | 2 | 101.57 |
|  | Ψ(.),*p*(site) | 105.69 | 16.18 | 0.0003 | 0.0003 | 2 | 101.69 |
|  | Ψ(.),*p*(foot,site) | 107.12 | 17.61 | 0.0001 | 0.0001 | 3 | 101.72 |
|  | Ψ(foot,site),*p*(.) | 109.26 | 19.75 | 0.0000 | 0.0001 | 3 | 103.26 |
|  | Ψ(site),*p*(foot) | 109.83 | 20.32 | 0.0000 | 0.0000 | 2 | 105.83 |
|  | Ψ(site),*p*(site) | 110.45 | 20.94 | 0.0000 | 0.0000 | 2 | 106.45 |
|  | Ψ(site),*p*(.) | 110.80 | 21.29 | 0.0000 | 0.0000 | 2 | 106.80 |
|  | Ψ(site),*p*(foot,site) | 111.83 | 22.32 | 0.0000 | 0.0000 | 3 | 106.83 |
|  | Ψ(foot),*p*(foot) | 256.22 | 166.71 | 0.0000 | 0.0000 | 2 | 252.22 |
|  |  |  |  |  |  |  |  |
| Species | Model | AIC | ΔAIC | AIC weights | Model Likelihood | K | -2*LogLike |
| *M. rotundata* | Ψ(site),*p*(foot,site) | 468.79 | 0.00 | 0.3536 | 1.0000 | 3 | 462.79 |
|  | Ψ(.),*p*(foot,site) | 469.58 | 0.79 | 0.2382 | 0.6737 | 3 | 463.58 |
|  | Ψ(foot),*p*(foot,site) | 470.36 | 1.57 | 0.1613 | 0.4561 | 3 | 464.36 |
|  | Ψ(foot,site),*p*(foot,site) | 470.55 | 1.76 | 0.1467 | 0.4148 | 4 | 462.55 |
|  | Ψ(site),*p*(site) | 474.03 | 5.24 | 0.0257 | 0.0728 | 2 | 470.03 |
|  | Ψ(foot),*p*(site) | 474.10 | 5.31 | 0.0249 | 0.0703 | 2 | 470.10 |
|  | Ψ(.),*p*(site) | 474.74 | 5.95 | 0.0180 | 0.0510 | 2 | 470.74 |
|  | Ψ(foot,site),*p*(site) | 475.26 | 6.47 | 0.0139 | 0.0394 | 3 | 469.26 |
|  | Ψ(foot),*p*(.) | 476.89 | 8.10 | 0.0062 | 0.0174 | 2 | 472.89 |
|  | Ψ(.),*p*(.) | 477.37 | 8.58 | 0.0048 | 0.0137 | 2 | 473.37 |
|  | Ψ(site),*p*(.) | 477.65 | 8.86 | 0.0042 | 0.0119 | 2 | 473.65 |
|  | Ψ(foot,site),*p*(.) | 478.68 | 9.89 | 0.0025 | 0.0071 | 3 | 472.68 |
|  | Ψ(.),*p*(foot) | 494.20 | 25.41 | 0.0000 | 0.0000 | 2 | 490.20 |
|  | Ψ(site),*p*(foot) | 494.45 | 25.66 | 0.0000 | 0.0000 | 2 | 490.45 |
|  | Ψ(foot,site),*p*(foot) | 496.05 | 27.26 | 0.0000 | 0.0000 | 3 | 490.05 |
|  | Ψ(foot),*p*(foot) | 500.30 | 31.51 | 0.0000 | 0.0000 | 2 | 496.30 |
|  |  |  |  |  |  |  |  |
| Species | Model | AIC | ΔAIC | AIC weights | Model Likelihood | K | -2*LogLike |
| *O. caerulescens* | Ψ(site),*p*(foot,site) | 474.12 | 0.00 | 0.2523 | 1.0000 | 3 | 468.12 |
|  | Ψ(.),*p*(foot,site) | 474.17 | 0.05 | 0.2461 | 0.9753 | 3 | 468.17 |
|  | Ψ(foot),*p*(foot,site) | 474.27 | 0.15 | 0.2341 | 0.9277 | 3 | 468.27 |
|  | Ψ(foot,site),*p*(foot,site) | 475.17 | 1.05 | 0.1493 | 0.5916 | 4 | 467.17 |
|  | Ψ(.),*p*(.) | 478.39 | 4.27 | 0.0298 | 0.1182 | 2 | 474.39 |
|  | Ψ(foot),*p*(.) | 479.03 | 4.91 | 0.0217 | 0.0859 | 2 | 475.03 |
|  | Ψ(site),*p*(.) | 479.04 | 4.92 | 0.0216 | 0.0854 | 2 | 475.04 |
|  | Ψ(site),*p*(site) | 480.36 | 6.24 | 0.0111 | 0.0442 | 2 | 476.36 |
|  | Ψ(.),*p*(site) | 480.45 | 6.33 | 0.0107 | 0.0422 | 2 | 476.45 |
|  | Ψ(foot),*p*(site) | 480.53 | 6.41 | 0.0102 | 0.0406 | 2 | 476.53 |
|  | Ψ(foot,site),*p*(.) | 480.85 | 6.73 | 0.0087 | 0.0346 | 3 | 474.85 |
|  | Ψ(foot,site),*p*(foot) | 482.20 | 8.08 | 0.0044 | 0.0176 | 3 | 476.20 |
|  | Ψ(site),*p*(foot) | 493.40 | 19.28 | 0.0000 | 0.0001 | 2 | 489.40 |
|  | Ψ(.),*p*(foot) | 494.10 | 19.98 | 0.0000 | 0.0000 | 2 | 490.10 |
|  | Ψ(foot,site),*p*(foot) | 495.39 | 21.27 | 0.0000 | 0.0000 | 3 | 489.39 |
|  | Ψ(foot),*p*(foot) | 499.51 | 25.39 | 0.0000 | 0.0000 | 2 | 495.51 |
|  |  |  |  |  |  |  |  |
| Species | Model | AIC | ΔAIC | AIC weights | Model Likelihood | K | -2*LogLike |
| *M. centuncularis* | Ψ(foot),*p*(.) | 287.52 | 0.00 | 0.3172 | 1.000 | 2 | 283.52 |
|  | Ψ(site),*p*(.) | 287.74 | 0.22 | 0.2842 | 0.8958 | 2 | 283.74 |
|  | Ψ(.),*p*(.) | 288.02 | 0.50 | 0.2470 | 0.7788 | 2 | 284.02 |
|  | Ψ(foot,site),*p*(.) | 289.38 | 1.86 | 0.1252 | 0.3946 | 3 | 283.38 |
|  | Ψ(.),*p*(site) | 293.63 | 6.11 | 0.0149 | 0.0471 | 2 | 289.63 |
|  | Ψ(.),*p*(foot,site) | 295.55 | 8.03 | 0.0057 | 0.0180 | 3 | 289.65 |
|  | Ψ(site),*p*(site) | 297.91 | 10.39 | 0.0018 | 0.0055 | 2 | 293.91 |
|  | Ψ(foot),*p*(site) | 297.99 | 10.47 | 0.0017 | 0.0053 | 2 | 293.99 |
|  | Ψ(site),*p*(foot, site) | 299.74 | 12.22 | 0.0007 | 0.0022 | 3 | 293.74 |
|  | Ψ(foot,site),*p*(site) | 299.78 | 12.26 | 0.0007 | 0.0022 | 3 | 293.78 |
|  | Ψ(foot),*p*(foot,site) | 299.82 | 12.30 | 0.0007 | 0.0021 | 3 | 293.82 |
|  | Ψ(foot,site),*p*(foot,site) | 301.74 | 14.22 | 0.0003 | 0.0008 | 4 | 294.74 |
|  | Ψ(.),*p*(foot) | 311.42 | 23.90 | 0.0000 | 0.0000 | 2 | 307.42 |
|  | Ψ(foot,site),*p*(foot) | 318.22 | 30.70 | 0.0000 | 0.0000 | 3 | 312.22 |
|  | Ψ(site),*p*(foot) | 319.75 | 32.23 | 0.0000 | 0.0000 | 2 | 315.75 |
|  | Ψ(foot),*p*(foot) | 332.91 | 45.39 | 0.0000 | 0.0000 | 2 | 328.91 |
|  |  |  |  |  |  |  |  |
