# Supplementary material for: The Bees among Us: Modelling Occupancy of Solitary Bees
Source: PLoS One. 2016 Dec 2;11(12):e0164764. doi: 10.1371/journal.pone.0164764 (PMC5135037; doi:10.1371/journal.pone.0164764)
Supplement: S2 Table — Significant differences calculated using the Z-score of building footprint. Asterisk indicates significant difference within species. (DOCX) [file pone.0164764.s004.docx]

|  |  | Ψ | | Abundance | | # Nesting tubes | |
| --- | --- | --- | --- | --- | --- | --- | --- |
|  | Species | F | p | F | p | F | p |
| Native | *M. campanulae* | 1.315 | 0.253 | 1.907 | **0.002*** | 1.158 | 0.254 |
|  | *M. pugnata* | 5.878 | **0.016*** | 0.188 | 1.000 | 0.123 | 1.000 |
|  | *O. pumila* | 0.610 | 0.436 | 1.986 | **0.001*** | 1.841 | **0.003*** |
| Introduced | *M. centuncularis* | 0.315 | 0.575 | 0.460 | 0.999 | 0.517 | 0.995 |
|  | *M. rotundata* | 0.306 | 0.581 | 0.773 | 0.847 | 0.930 | 0.605 |
|  | *O. caerulescens* | 0.257 | 0.613 | 0.482 | 0.998 | 0.899 | 0.658 |

**S2 Table. Statistical output from comparisons of occupancy probability using the model equation Ψ(site),*p*(.) for each bee species,** **and the proportion of building footprint within a 300m radius around each site.** Significant differences calculated using the Z-score of building footprint. Asterisk indicates significant difference within species.
